# Effectiveness, costs and cost-effectiveness of chiropractic care and physiotherapy compared with information and advice in the treatment of non-specific chronic low back pain: study protocol for a randomised controlled trial

**DOI:** 10.1186/s13063-017-2351-3

**Published:** 2017-12-22

**Authors:** Filip Gedin, Martin Skeppholm, Kristina Burström, Vibeke Sparring, Mesfin Tessma, Niklas Zethraeus

**Affiliations:** 1 Karolinska Institutet, Department of Learning, Informatics, Management and Ethics (LIME), Medical Management Centre, Health Economics and Economic Evaluation Research Group, Tomtebodavägen 18A, 17177 Stockholm, Sweden; 2 Karolinska Institutet, Department of Learning, Informatics, Management and Ethics (LIME), Medical Management Centre, Health Economics and Economic Evaluation Research Group, Tomtebodavägen 18A, 17177 Stockholm, Sweden; 3 Ryggkirurgiskt Centrum Stockholm AB, Sophiahemmets sjukhus, Box 5605, 11485 Stockholm, Sweden; 4 Karolinska Institutet, Department of Learning, Informatics, Management and Ethics (LIME), Stockholm Centre for Healthcare Ethics, Health Outcomes and Economic Evaluation Research Group, Tomtebodavägen 18A, 17177 Stockholm, Sweden; 5 Karolinska Institutet, Department of Public Health Sciences, Equity and Health Policy Research Group, Tomtebodavägen 18A, 17177 Stockholm, Sweden; 6 Stockholm County Council, Health Care Services, Tomtebodavägen 18A, 17177 Stockholm, Sweden; 7 Karolinska Institutet, Department of Learning, Informatics, Management and Ethics (LIME), Medical Management Centre, Tomtebodavägen 18A, 17177 Stockholm, Sweden; 8 Karolinska Institutet, Department of Learning, Informatics, Management and Ethics (LIME), Medical Statistics Unit, Tomtebodavägen 18A, 17177 Stockholm, Sweden

**Keywords:** Chiropractic, Chronic low back pain, Cost, Effectiveness, Exercise, Manipulation, Physiotherapy

## Abstract

**Background:**

Low back pain is a global public health problem and a leading cause of disability all over the world. The lifetime prevalence of low back pain is 70–80% and a significant proportion of people affected develop chronic low back pain (CLBP). Besides a severe negative impact on people’s health and health-related quality of life, CLBP is associated with substantial costs for society. Medical costs for the management of CLBP and costs for production losses due to absenteeism from work are sizeable. Pharmaceuticals, physical activity, manipulation, and multidisciplinary rehabilitation interventions are examples of widely used treatments for CLBP. However, the scientific basis to recommend the use of one treatment over another is limited and more research is needed to study the effects, costs and cost-effectiveness of treatments for CLBP in clinical practice.

The aim of the study is to evaluate the effectiveness (back pain-related functional limitation, back pain intensity, general health, health-related quality of life, and working status), costs (medical costs and costs for production losses) and cost-effectiveness of chiropractic care and physiotherapy when added to information and advice in the treatment of patients with non-specific CLBP in Sweden.

**Methods/design:**

This is a pragmatic randomised controlled trial, where participants are recruited through six primary care rehabilitation units (PCRUs) in Stockholm County Council, Sweden. Individuals with non-specific CLBP are individually randomised to one of four treatment groups: ‘information and advice’; ‘physiotherapy, and information and advice’; ‘chiropractic care, and information and advice’; or ‘chiropractic care, physiotherapy, and information and advice’. A sample size of 600 participants will be recruited during a period of 33 months. A computer-based questionnaire is used to collect data on back pain-related functional limitation (Oswestry Disability Index), pain intensity (Numeric Rating Scale), general health (self-rated health), health-related quality of life (EQ-5D-3L), and working status (measured as percentage of full-time work). Data will be collected at baseline, and at 3, 6, and 12 months after baseline.

**Discussion:**

The results from our study should be considered when producing evidence-based guidelines and recommendations on which treatment strategies to use for CLBP.

**Trial registration:**

ISRCTN registry, ID: ISRCTN15830360. Registered prospectively on 2 February 2017.

**Electronic supplementary material:**

The online version of this article (doi:10.1186/s13063-017-2351-3) contains supplementary material, which is available to authorized users.

## Background

Low back pain is one of the leading causes of disability across the world [1]. In the World Health Organisation (WHO) study on the global burden of disease, low back pain accounted for close to 11% of the total number of years lived in disability (YLD), making low back pain the leading cause of YLD in the world [1]. The total cost of low back pain to society was estimated at €1 860 million in Sweden in 2001 [2]. The indirect costs due to lost productivity were sizeable and accounted for 84% of the total cost [2]. In Sweden, low back pain is the most common reason for granting sick leave for patients with musculoskeletal disorders [3]. The lifetime prevalence of low back pain is 70–80% and a significant proportion of patients (10–20%) develop chronic low back pain (CLBP) lasting for at least 3 months [4, 5]. CLBP is associated with long-term pain, impaired function, and a severe negative impact on people’s health and health-related quality of life (HRQoL) [5]. In addition, CLBP is associated with substantial costs arising from within, but also outside, the health care sector. It is estimated that the total yearly cost for a patient with CLBP is SEK 227,000 within primary care [6, 7]. The indirect costs for production losses due to lost work days stood for about 85% of the total costs [6]. Health care systems should prioritise identifying effective and cost-effective treatment strategies to decrease the disease burden of CLBP.

A wide range of treatment options exist in the treatment and management of CLBP. Different pharmacological and non-pharmacological treatments, like physical activity, manipulation, and multidisciplinary rehabilitation interventions, are widely used alone or in combination [8, 9]. According to a survey published in a report by the Swedish Agency for Health Technology Assessment and Assessment of Social Services (SBU) [7], treatments for acute back pain in Sweden are often given by physiotherapists, chiropractors or naprapaths. While all of these occupational groups provide information and advice to stay active, they usually combine different interventions [7]. Physiotherapists typically also use different types of training and exercise in the treatment of acute back pain, than those used by chiropractors. For example, in the survey by SBU [7], 69% of the physiotherapists stated that they frequently used ‘circulation training’ as compared to 36% of chiropractors [7]. Chiropractors and naprapaths also use forms of manual therapies that physiotherapists do not, in general, use. The results in the survey showed that 13% of the physiotherapists regularly used spinal manipulation whereas the corresponding figure for chiropractors was 96% [7]. The report by SBU (2016) probably also reflects what occupational groups and treatments are involved in the treatment of patients with CLBP [7]. Our study will further increase the knowledge on what treatments physiotherapists and chiropractors currently use for the treatment of CLBP in Sweden.

The scientific basis to recommend the use of one treatment alternative over another is inadequate or limited and, therefore, more research is needed to study the effectiveness [9–11], and cost-effectiveness [12–15] of treatments for CLBP. According to findings in systematic literature reviews, there is high-quality evidence that spinal manipulation is as effective as other therapies (exercise therapy, standard medical care or physiotherapy) in reducing pain and improving function [10]. Furthermore, there is low-to-moderate evidence for (motor control) exercise to be clinically important, when compared to minimal intervention, and there is moderate-to-good evidence that motor control exercises have similar outcomes as other exercises and manual therapies [11]. There is moderate evidence that multidisciplinary treatments are more likely to have less pain and disability for patients compared to patients receiving usual care or physiotherapy [9]. However, SBU (2015) stated in a comment that the results in Kamper et al. (2015) were uncertain and maybe due to publication bias [9, 16].

In a previous pragmatic randomised controlled trial (RCT) in Sweden, Skargren et al. [12] compared the outcomes and costs of chiropractic care and physiotherapy as a primary treatment for patients with back and neck pain over a 1-year follow-up [14]. No difference in costs and outcomes were demonstrated for the total population. However, subgroup analyses showed that patients with acute, uncomplicated back pain gained more from chiropractic care at a similar cost, whereas patients with chronic back pain gained more from physiotherapy with a slightly reduced cost [12]. Niemisto et al. (2003) found that a combined intervention (advice, physiotherapy, and spinal manipulation) was more effective in reducing pain and disability than a physician consultation alone [13]. There were no significant differences in costs between the treatment groups [13]. In a follow-up study 2 years later, it was indicated that a physician consultation was cost-effective [14]. A cost-effectiveness study conducted alongside a RCT, concluded that spinal manipulation was a cost-effective addition to ‘best care’ for back pain in general practice, but also found that manipulation alone probably was cost-effective compared with manipulation followed by exercise in a UK setting [15].

With limited health care resources and stretched health care budgets, it is important to strive for efficient use of scarce resources [17]. Economic evaluation is a method for assessing costs and effectiveness of alternative ways of allocating resources to assist decisions aimed at improving efficiency [17, 18]. It also constitutes an important input for guiding clinical decisions. To be useful for decision-makers as an input to improve decision-making on the societal efficiency of the allocation of health care resources, it is important that the economic evaluation is based on a societal perspective, including costs both within (e.g. costs for pharmaceuticals, and costs for hospital and ambulatory care) and outside (e.g. indirect costs for decreased labour production) the health care system [18, 19]. In economic evaluations it is common to carry out cost-effectiveness analyses, where costs are measured in monetary terms and effectiveness in non-monetary terms. In this type of analysis, where the aim is to maximise the health gains for a specific cost constraint, it is crucial to use an outcome measure that combines effects on quality and quantity of life into one measure. The most commonly used measure of this sort is the quality-adjusted life year (QALY) [17]. This type of outcome measure is also recommended by, e.g. the National Institute for Health and Care Excellence (NICE) in England and Wales, and by the Dental and Pharmaceutical Benefits Agency in Sweden [20, 21].

Economic evaluation can be used to guide resource allocation decisions on which (if any) treatments for CLBP should be recommended. To achieve valid and reliable cost-effectiveness results in the area of CLBP the economic evaluation should be conducted alongside a RCT designed to reflect the clinical situation to which the decision is being applied and with a sufficient follow-up period carried out in well-defined study populations [22, 23]. This opens up the possibility to perform the economic evaluation based on data characterised by both high internal and external validity. A limitation of conducting an economic evaluation alongside a RCT is that the sample size is usually determined based on primary clinical endpoints in the trial. This implies that the power to detect different hypothesised cost-effectiveness results may be low. However, by using the net benefit approach, it is possible to investigate the power in a cost-effectiveness study that is implied by the given sample size in the RCT [24]. An advantage of using a so-called pragmatic RCT as a basis for the economic evaluation is that patient-level trial data provide an unbiased estimate of the effectiveness of interventions as reflected in clinical practice [23]. In addition, the RCT provides an opportunity for collecting data on the resources used to estimate costs and cost-effectiveness [19].

Currently, there are no clinical guidelines in Sweden on which treatment option should be used for CLBP [25]. This reflects a lack of knowledge on the effectiveness and cost-effectiveness of different treatment strategies for CLBP in a Swedish context [25, 26]. Thus, it is important to increase this knowledge. This is central for government agencies and county councils to be able to produce evidence-based guidelines and recommendations on which treatment strategies to use for CLBP.

In Sweden, patients with low back pain are often treated by either chiropractors or physiotherapists. Sometimes patients are treated by chiropractors and physiotherapists in collaboration. Besides providing general information and advice, these occupational groups usually recommend exercise (mostly recommended by physiotherapists) and manual therapies (mostly recommended by chiropractors) [7]. These treatment alternatives are also recommended in guidelines in different parts of the world [27]. As noted above, although a wide range of treatments exist for CLBP, the scientific basis to recommend the use of one treatment alternative over another is limited. There is, therefore, a need for well-designed clinical trials investigating both the effectiveness and cost-effectiveness of treatments for CLBP. Cost-effectiveness studies may provide valuable information on whether it is good value for money to use treatments and examinations provided by chiropractors and/or physiotherapists, in addition to only providing information and advice. Information both about the cost-effectiveness and effectiveness of treatments for CLBP is crucial for decisions aiming at improving the efficiency in the allocation of limited resources for the treatment of CLBP. The results from our study will improve the basis for health care decision-making and policy recommendations and should be considered when producing and updating evidence-based guidelines and recommendations on which treatment strategies to use for CLBP.

The aim of the study is to evaluate the effectiveness (back pain-related functional limitation, back pain intensity, general health, HRQoL, and working status), costs (medical costs and costs for production losses) and cost-effectiveness of chiropractic care and physiotherapy when added to information and advice in the treatment of patients with non-specific CLBP in Sweden.

Research questions:

1. How do chiropractic care and physiotherapy, chiropractic care, or physiotherapy, when added to information and advice, impact outcomes (back pain-related functional limitation, back pain intensity, general health, HRQoL, and working status) as compared with only information and advice?
2. Which (if any) of chiropractic care and physiotherapy, chiropractic care, or physiotherapy, when added to information and advice, is a cost-effective treatment strategy when compared to each other, and when compared with only information and advice?

## Methods/design

### Design

This is a multicentre, pragmatic RCT, approved by the Regional Ethical Review Board, Stockholm (Dnr: 2016/1318-31-31), and prospectively registered in the ISRCTN Registry (2017-02-20: ISRCTN15830360). The reasons for a pragmatic design are to reflect clinical practice and to limit per-protocol effects on the outcomes [23]. Each participant’s treatment is at the discretion of the individual chiropractor and/or physiotherapist. In Sweden, there are no official guidelines that define what those treatments typically include or how many sessions are standard. This study protocol has followed the Standard Protocol Items: Recommendations for Interventional Trials (SPIRIT) Guidelines (Additional file 1). The trial will be conducted and reported according to the reporting of pragmatic trials: an extension of the Consolidated Standards of Reporting Trials (CONSORT) Statement [28].

### Setting

The study will be conducted at six primary care rehabilitation units (PCRUs) in Stockholm County Council, Sweden. If needed, additional units will be included. The operations manager for each PCRU has signed a written certificate to assure that they will provide sufficient resources to both carry out the study and guarantee the safety of the participants. The PCRUs are reimbursed through the Stockholm County Council. Twelve PCRUs in the Stockholm County Council were initially informed about the study by email and invited to participate. For each of the six PCRUs interested in participating, an information meeting was scheduled. Before the start of the study each PCRU was informed about the study methods in more detail and given the opportunity to give feedback on how to improve the efficiency in the recruitment of participants.

### Characteristics of participants

The study includes individuals aged between 18 to 60 years, who are willing to participate in any of the included treatment groups (‘information and advice’; ‘physiotherapy, and information and advice’; ‘chiropractic care, and information and advice’; or ‘chiropractic care, physiotherapy, and information and advice’). All patients give written informed consent. The inclusion and exclusion of study participants is done to mimic the clinical praxis of Swedish primary health care, where the first patient contact usually takes place by phone. Inclusion criteria are: pain located below the costal margin and above the inferior gluteal folds, reoccurring low back pain for at least 3 months, age between 18 to 60 years, can stand or walk independently, and being Swedish speaking and literate. Exclusion criteria are: pain attributable to a known specific pathology (e.g. pain related to tumours, fractures, or fibromyalgia), lack of written informed consent, pregnancy or less than 6 months postpartum or post weaning, been treated for low back pain by a chiropractor and/or physiotherapist in the previous 1 month.

### Recruitment procedures

All participants are recruited through the PCRUs. Individuals with low back pain who contact the PCRU by phone are informed about the study and invited to participate. For individuals who are willing to participate (orally informed consent is given), and who meet the eligibility criteria (assessed by a primary health care staff member), a first (baseline) visit is scheduled at the PCRU. Information about the study will also be sent by email to the participants, after the phone contact and before the first PCRU visit. During the first visit to the PCRU, the participants sign a letter of informed consent before the treatment starts. At each PCRU two study coordinators (chiropractor and physiotherapist) are responsible for the enrolment of participants. Being a pragmatic RCT, there are no criteria for discontinuing or modifying allocated interventions, or improving adherence for a given trial participant. There is also no relevant concomitant care or intervention permitted or prohibited during the trial. The patients have the option to withdraw from the study at any point in time.

The recruitment of participants needs to be carefully monitored for each PCRU without any interaction between researchers and potential study participants. Throughout the study, the study coordinators at each PCRU will be contacted by the first author (FG) on a weekly basis about the enrolment of participants and the recruitment rate. During the start of the study (first 4 weeks) FG also assesses whether the information made available to each PCRU is clear regarding study methods and recruitment procedures. If needed, the information about study methods and recruitment procedures will be updated and clarified. After potential amendments, all PCRUs will continue the recruitment of participants according to the updated procedures. Additionally, FG will meet all the study coordinators on a monthly basis to review and gain feedback on the enrolment procedures and the recruitment rate.

### Randomisation

**Fig. 1:**
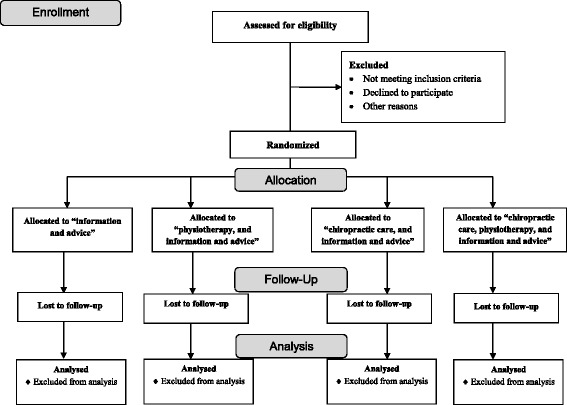
Study trial profile and randomisation

Each PCRU will continue to include subjects until the required total number of subjects (*n* = 600) is achieved (Fig. 1). All patients who give informed consent and who fulfil the eligibility criteria will be randomised. Participants are randomised to one of four treatments: ‘information and advice’; ‘physiotherapy, and information and advice’; ‘chiropractic care, and information and advice’; or ‘chiropractic care, physiotherapy, and information and advice’. A computer-generated, block randomisation list is produced to allocate participants to the treatment wings. The randomisation list and the allocation sequence are generated by a statistician who is not involved in the study. The sequence is concealed from the researchers involved in enrolling and assessing participants by using sequentially numbered, opaque, sealed envelopes. All researchers involved in the study will be blinded to block size(s) and randomisation list. Thus, the researchers do not know the block size(s) or if there is only one block size or different block sizes. This information (about block size(s) and randomisation list) is only known by the independent statistician who is not involved in the trial. The randomisation list will be in the possession of the statistician for the duration of the study. Envelopes will only be opened when it is time to allocate the intervention. The primary health care staff member opens the envelope during the phone call (but does not reveal the allocation to the participant), and schedules a time for the first visit. During the first visit at the PCRU, the health care provider (chiropractor and/or physiotherapist) will reveal which treatment group the participant has been randomised to.

### Blinding

The study is a pragmatic trial where blinding is neither possible nor desirable. However, outcome assessors and data analysts will be blinded to group allocation.

### Study treatments

During the first (baseline) visit all participants will meet a chiropractor and/or a physiotherapist for an initial examination which includes diagnosis and the medical history of the patient. The participants are randomised to one of four treatment groups:

1. *Information and advice*: participants are given oral and written information on how to manage non-specific CLBP and advice about the importance of staying active and avoiding rest. Each participant is given a booklet with information on how to stay active [7, 29]
2. *Physiotherapy, and information and advice*: the treatment primarily involves training and exercise such as stabilisation training, functional training, mobility training and postural control [7]. The duration, number and content of the treatment is at the discretion of the physiotherapist. Participants are also given information, advice, and a booklet [29]
3. *Chiropractic care and information and advice*: the treatment primarily involves spinal manipulation defined as a high-velocity, low-amplitude movement at the limit of joint range that takes the joint beyond the passive range of movement [7]. The duration, number, and content of the treatment is at the discretion of the chiropractor. Participants are also given information, advice, and a booklet [29]
4. *Chiropractic care, physiotherapy, and information, and advice*: the treatment primarily involves a combination of spinal manipulation (defined as a high-velocity, low-amplitude movement at the limit of joint range that takes the joint beyond the passive range of movement) and training and exercise (e.g. stabilisation training, functional training, mobility training and postural control) [7]. The duration, number, and content of the treatment is at the discretion of the chiropractor and physiotherapist. Participants are also given information, advice, and a booklet [29]

### Outcome measurements

A computer-based, self-reported patient questionnaire is used to collect data on back pain-related functional limitation, pain intensity, general health, HRQoL, and working status. Back pain-related functional limitation is measured by using the Oswestry Disability Index (ODI), pain intensity by using the Numeric Rating Scale (NRS), general health by using the general self-rated health (SRH) question and HRQoL is measured by the EuroQol 5 Dimensions 3 Levels (EQ-5D-3L) instrument [30–33].

#### Primary outcome measure

##### Oswestry Disability Index (ODI) − A measure of back pain-related functional limitation

The ODI is a condition-specific instrument that measures back pain-related functional limitation [30]. It consists of ten questions where each question has six response choices and the value of each question ranges from 0 (no problems) to 5 (worst imaginable problems). A total ODI score is computed as the sum of scores on each question, and the total score of ODI varies between 0 (no problems) and 50 (worst imaginable problems). The total ODI score is then normalised to 0–100, by multiplying the (unadjusted) total score by 2. The ODI normalised score, based on the ODI outcome measure at 6 months, is our primary outcome measure [12]. The ODI has a high reliability, validity, and responsiveness, and is easy to administrate and is sensible to find clinical changes in treatment of patients with CLBP [34, 35].

#### Secondary outcome measures

##### Numeric Rating Scale (NRS) – A measure of pain intensity

The NRS is an 11-grade scale ranging from 0 (no pain) to 10 (worst pain imaginable). The NRS is often used in clinical trials and by clinicians to measure self-reported pain intensity. It is easy to administrate and has a high responsiveness [31]. Each participant is asked to state their currently experienced pain using the NRS.

##### Self-rated health (SRH) – A measure of general health

As a measure of general health, a self-rated health (SRH) question will be used. The SRH question is phrased as: ‘In your opinion, what is your health status? Is it very good, good, fair, bad, or very bad?’ We use a pre-scored Swedish experienced-based value set (as presented in the Online Resource with supplementary material to Burström et al. 2014 [32]), to transform SRH severity levels into quality-of-life (time-trade off) values.

##### EQ-5D-3L – A measure of health-related quality of life

The EQ-5D-3L is a generic instrument measuring HRQoL. The EQ-5D-3L is a questionnaire consisting of five dimensions with three severity levels and a Visual Analogue Scale (VAS) ranging from 100 (best imaginable health state) to 0 (worst imaginable health state) [33]. The dimensions and levels result in 243 possible EQ-5D health states. Each health state can be assigned a value based on country-specific value sets. We use the Swedish experienced-based value set to transform EQ-5D health states into HRQoL (time-trade off) values [32]. The EQ-5D-3L is a valid instrument among patients with pain [31, 36]. The EQ-5D-3L is commonly used in Swedish health care and enables cross-sectional comparisons.

##### A measure of working status

The study participants will be asked to answer questions about current working status and number of hours absent from work due to illness during the past 7 days. For each participant, working status is defined as self-reported percentage of full-time work.

#### Exploratory outcome measures

##### Costs and quality-adjusted life years (QALYs)

We will analyse the difference in costs during 12 months after the randomisation between the different treatment groups. Both direct and indirect costs will be estimated. Direct costs include costs for pharmaceuticals, health care visits, clinical examinations, and hospital days. Information about each participant’s resource consumption is collected by using a computer-based questionnaire. At each follow-up occasion (at baseline, and at 3, 6, and 12 months after baseline) the participant is asked to recall their resource consumption during the last 3 months (quantities of resource consumption is, e.g. number of prescriptions, number of visits to health care providers, number of clinical examinations, and number of hospital days). To estimate the direct costs the quantities of resource consumption is multiplied by the unit costs. Unit costs of pharmaceuticals are collected from the price database available at TLV and unit costs for health care visits will be based on prices in the Stockholm County Council for primary health care [37, 38]. Unit costs for clinical examinations and hospital days will be based on unit costs in the Stockholm County Council and in the southern part of Sweden (Region Skåne) [39].

The indirect costs refer to costs of changes in productivity (labour production) [40]. Data on changes in productivity while being at work (presenteeism) is not collected in the study. The working status of each participant, defined as the percentage of full-time work, was recorded at baseline and at 3, 6, and 12 months after baseline. The working status is estimated as the percentage of full-time work for each participant. The labour production value was estimated for the period from the first visit (at baseline) until 3 months, and for 4 to 6 months, and for 7 to 12 months. The average working status during the first of these periods (0–3 months) was assumed to be an average of the working status at baseline and 3 months. The average working status during the other periods (4–6 months and 7–12 months) was calculated equivalently. To estimate the value of production for the first 12 months the average working status during the first 12 months will be multiplied by the average value of labour production of a Swedish worker [40, 41].

To estimate the number of QALYs for each treatment the quality-of-life values based on EQ-5D-3L will be used. The average number of QALYs during 12 months is calculated as the area under the curve during 12 months. A treatment is defined as cost-effective if the treatment is less costly while providing the same or better health outcomes, or if the added cost of the treatment is reasonable in comparison to the health outcomes obtained, in which case the incremental cost per gained QALY is estimated. An intervention is in the latter case defined as cost-effective if the cost per QALY gained is below or equal to a threshold value corresponding to how much society is willing to pay in order to gain a QALY. Different threshold values for a QALY have been used in cost-effectiveness studies and the value has usually been set to around US$50,000 (corresponding to approximately SEK 400,000 using the average exchange rate during 2016 of 1 US$ = SEK 8.6). However, Neumann et al. (2014) argue for using a threshold value of either US$100,000 or 150,000 for a QALY and that a threshold value of US$50,000 should be interpreted as a lower boundary [42]. If the threshold value of a QALY is derived from the literature on the value of a statistical life, even higher threshold values are derived (US$100,000–400,000) [42, 43]. In Sweden, the Swedish Transport Administration (STA) uses a value of a statistical life of SEK 24 million in cost-benefit analysis of road investments. Using this figure, a value per QALY gained of about SEK 950,000 can be derived (US$110,000). In our study, we define an intervention to be cost-effective if the cost per gained QALY is equal to or below SEK 900,000 (US$100,000).

The cost-effectiveness analysis will be reported in accordance with the Consolidated Health Economic Evaluation Reporting Standards (CHEERS) [44] and good research practices on how to conduct cost-effectiveness analysis alongside clinical trials [21]. Unit costs, data sources, and mean use of health care resources will be reported for each treatment. Mean costs for each treatment will be presented as well as incremental costs and effectiveness.

### Data monitoring

A Data Monitoring Committee (DMC) will not be established in this trial because there are no unusual high safety concerns related to our included trial participants. The treatments in this trial do not differ from the standard primary care accepted by the Swedish Association of Local Authorities and Regions and the risk of harm for trial participants is considered low. No interim analysis will be performed, and we do not plan to collect and report adverse events and other unintended effects. The recruitment of patients will be carefully monitored during the whole study by one of the study researchers (FG), who will be in contact with the study coordinators of each PCRU. If needed, measures will be taken and adjustments made to increase the speed of the recruitment rate. FG will also meet all the study coordinators each month to review the enrolment procedures of participants and the recruitment rate.

### Data collection

Data will be collected at baseline (after randomisation and before the treatment starts), and at follow-up, 3, 6, and 12 months after baseline. Each participant fills out a computer-based questionnaire at each measurement occasion. The day before each follow-up occasion, each participant is sent a reminder by email to fill out the questionnaire. If needed, a second and a third reminder is sent 2 and 7 days after the follow-up occasion. All data will be obtained from the computer-based questionnaire and from the chiropractor and/or physiotherapist reporting the number and content of treatments.

#### Baseline

Baseline data will be collected during the first visit or at 1–4 days before the first visit at the PCRU. Data on individual characteristics (age, sex, education, smoking status, physical activity, use of pain killers, and pain duration), outcome measures (back pain-related functional limitation, pain intensity, general health, HRQoL, working status) and resource consumption (pharmaceuticals, health care visits, clinical examinations, and hospital days) are collected.

#### Follow-up at 3, 6, and 12 months

At 3, 6, and 12 months after baseline data on outcome measures (back pain-related functional limitation, pain intensity, general health, HRQoL, working status) and resource consumption (pharmaceuticals, health care visits, clinical examinations, and hospital days) are collected. At each follow-up occasion (and at baseline) the participant is asked to recall their resource consumption during the last 3 months. Data on physical activity is also collected at follow-up.

### Timeline

**Fig. 2:**
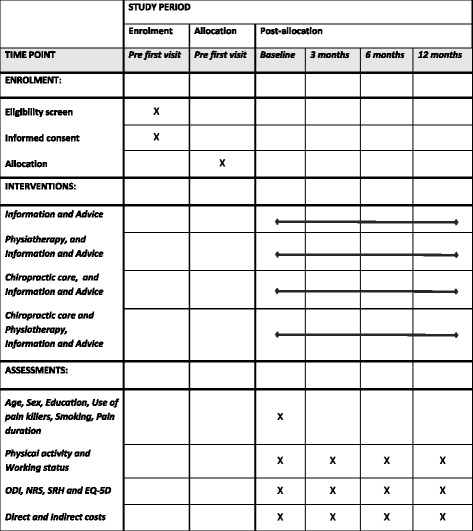
Schedule of enrolment, interventions, and assessments

The recruitment of patients started on 1 April 2017 and is expected to be finalised by 31 December 2019. All data for all follow-up occasions is expected to be collected by 31 December 2020. The data analysis, writing of scientific manuscripts and submissions to peer-reviewed scientific journals will be carried out during 2021 and 2022. A summary of the study outline is given in Fig. 2.

#### Required sample size

A change of 10 percentage points on the ODI scale is usually defined as the minimal clinically important difference (MCID) [45]. To detect a reduction of 10 percentage points (SD of 15) in the ODI score, which is in agreement with the study of Davidson et al. [45] and Hägg et al. [46], with a two-sided 5% significance level and a power of 80%, a sample size of 150 patients per group will be necessary, given an anticipated dropout rate of 20%. Each PCRU will recruit subjects until the required number is achieved. The enrolment period will extend over 33 months. The projected sample size will provide sufficient statistical power for linear mixed regression and Generalised Estimating Equation (GEE) models considering compound symmetry of the covariance structure and the number of repeated measures [47]. Further, a change of 10–30 percentage points in the NRS scale is usually defined as a MCID [48, 49]. To detect a reduction of 10 percentage points in the NRS, with a two-sided 5% significance level and a power of 80%, a sample size of 146 patients per group will be necessary, given an anticipated dropout rate of 20%.

### Statistical analysis

The main analysis will be conducted as an intention-to-treat (ITT) analysis for all participants that are included in the study [50]. Pattern of missing data and dropout will be examined and if appropriate multiple imputations will be used based on the nature of the missing data. The primary analysis is to evaluate between group differences in change on ODI scores at 6 months. All statistical tests are carried out at the 5% significance level (two-sided).

#### Primary outcome measure

The primary analysis is to evaluate between-group differences in percentage point change in ODI scores at 6 months compared to baseline. One-way analysis of variance (ANOVA) will be used to evaluate the between-group difference for the primary outcome variable ODI at 6 months. A Levine’s test will be employed to check the assumption of equality of variance for the ANOVA. Assumption of normality will be checked by using skewness and normal probability plot. In case of violation of the normality assumption, we will use the non-parametric Kruskal-Wallis H test which does not require the assumption of normality. If the assumption of homogeneity of variance is violated we will use, the Welch or the Brown and Forsythe test. If the overall ANOVA is significant, we will perform further analysis using a Tukey or Games-Howell post hoc pairwise comparison. We will use Games-Howell if there is a violation of the equality of variance assumption. To evaluate effect size, we will present the difference between means, 95% confidence interval and the partial eta-squared (*η* ^2^).

#### Additional analysis for percentage point change in ODI

Two additional analyses will be carried out for the primary outcome variable ODI at 6 months. First, an adjusted analysis will be used to control for baseline variables (age, sex, education, smoking status, physical activity, use of pain killers). This multiple linear regression analysis will adjust for potential predictor variables which might reasonably be expected to be predictive of favourable outcomes. We will perform residual analysis to assess model assumptions and goodness-of-fit. Secondly, a subgroup analysis will be performed to detect differences between groups of patients with an ODI normalised score at baseline either less than 40% or equal to or greater than 40% [12].

#### Secondary outcome measures

Additional analysis of ODI will be done to evaluate the percentage point change in ODI scores and improvement at 3 and 12 months, respectively. Since the trial involves repeated measures data, a linear mixed model will also be used to analyse the secondary outcome measure. To account for repeated measures, to model within-subject variance, and to handle correlated data of continuous variables we will use a linear mixed model. An interaction term will be introduced in the model to examine heterogeneity effect.

The effect of treatment will be evaluated separately for each outcome variable (both primary and secondary) by using a linear mixed model. For binary and ordinal outcome variables a GEE will be employed. The latter model will be used to evaluate the effect of treatment on physical activity at 3, 6, and 12 months.

#### Exploratory outcome measures

Uncertainty will be reported by estimating 95% confidence intervals for the mean value of costs and effectiveness in each group, and for the difference in mean value of costs and effectiveness between groups. Non-parametric bootstrap analysis will be carried out to estimate the sampling distribution of the ICER estimator in the cost-effectiveness plane (where each treatment is compared with information and advice) [51]. To reflect uncertainty in the threshold value for a QALY, cost-effectiveness acceptability curves will be computed, where the probability of cost-effectiveness is presented for different threshold values (from SEK 0 to 1,500,000). Cost-effectiveness acceptability curves will be computed based on parametric and non-parametric bootstrap methods using the net benefit approach [51]. Sensitivity analysis will also be carried out where the unit costs will be changed (±50%), and where QALYs, instead of being based on EQ-5D-3L, will be estimated based on SRH. In another sensitivity analysis, the indirect costs will be estimated based on number of hours absent from work during the past 7 days. The average number of hours absent from work during the first 3 months was assumed to be an average of the hours absent from work at baseline and at 3 months, whereas the average number hours absent from work during the other periods (4–6 months and 7–12 months) was calculated equivalently. To estimate the loss in production due to absence from work for the first 12 months, the average number of hours absent from work during each period will be multiplied by the average per hour value of labour production for a Swedish worker.

## Communication of trial results

The project is expected to produce at least two scientific papers to be published in international scientific journals. The first paper will summarise all the results on the effect of the included treatments on outcomes. The second paper includes the cost-effectiveness results where the costs of the different treatments are related to the effectiveness and to the changes in QALYs.

The scientific papers emanating from the project will be submitted to international scientific journals during 2021 and 2022. In addition to international scientific publishing, the research group plans to publish the main results of the project in medical journals in Sweden. The plan is also to present the studies at national and international scientific conferences such as the Swedish Health Economics Association and the Spine Society.

## Discussion

In this pragmatic RCT we investigate the effectiveness and cost-effectiveness of chiropractic care and/or physiotherapy, when added to information and advice. The strengths of the trial include the use of a pragmatic RCT to reflect clinical practice, the conduct of an economic evaluation alongside a RCT, the use of standardised health outcome measures and a randomisation process with the researchers blinded for treatment group allocation, and the recruitment of large study population with a sufficient power to detect clinically important differences between groups.

A limitation of the study is the difficulty in blinding patients as to which treatment alternative they are allocated, which could lead to an expectation bias that may influence the results. An expectation bias may work in both directions depending on whether patient expectations and beliefs are positive (placebo effect) or negative (nocebo effect). We have not included any questions to measure patient expectations and beliefs before the treatment starts, which implies that we cannot estimate the potential expectation bias. On the other hand, which is a strength of the study, the results from our pragmatic RCT will reflect real effects for patients with CLBP in a primary care setting. Some patients do not always feel that they will get the treatment they would prefer, which may also affect the outcome of the treatment in clinical practice. Another limitation is the follow-up period in the trial, which is 1 year. Ideally, we would like to have a 2-year follow-up to be able to examine the effectiveness and cost-effectiveness of chiropractic care and physiotherapy in the long run.

CLBP is a severe public health problem associated with substantial costs for society. Although a wide range of treatment options exist for CLBP, the scientific basis to recommend the use of one treatment alternative over another is limited. The results from our study will improve the basis for health care decision-making and policy recommendations on how to use limited health care resources to improve health for people suffering from CLBP in Sweden. Our findings should be considered when producing and updating evidence-based guidelines and recommendations on which treatment strategies to use for CLBP.

## Trial status

Protocol version 1: 9 July 2017. Recruitment started 1 April 2017 and is expected to be finalised by 31 December 2019.

## Additional file

Additional file 1: SPIRIT Checklist: recommended items to address in a clinical trial protocol. (PDF 130 kb)

## Abbreviations

CLBP: Chronic low back pain
EQ-5D-3L: EuroQol 5 Dimensions 3 Levels
NRS: Numeric Rating Scale
ODI: Oswestry Disability Index
PCRU: Primary Care Rehabilitation Unit
QALY: Quality-adjusted life year
SRH: Self-rated health
